# Robust portfolio optimization for banking foundations: a CVaR approach for asset allocation with mandatory constraints

**DOI:** 10.1007/s10100-022-00821-5

**Published:** 2022-10-28

**Authors:** Maria Cristina Arcuri, Gino Gandolfi, Fabrizio Laurini

**Affiliations:** 1 Department of Economics and Management, University of Parma, Via J.F. Kennedy 6, 43125 Parma, Italy; 2 Knowledge Group Banking and Insurance, SDA Bocconi School of Management, Milan, Italy

**Keywords:** Foundations of banking origin, Robust C-VaR, Robust portfolio optimization, C44, C61, G11, G23

## Abstract

This paper focuses on an innovative asset allocation strategy for risk averse investors who operate on very long-time horizons, such as endowments and the Italian foundations of banking origin (FBOs). FBOs play a pivotal role in supporting economic, financial and sustainable growth in the long term. In the search for a model which optimizes FBO portfolio choices in the light of regulatory constraints on their sizeable investable portfolio, we highlight the risk-adjusted performances obtained using a robust conditional VaR (R-CVaR) approach—assuming different risk profiles—which corrects some of the Markowitz approach pitfalls and accounts for tail risk. We compare the two models using a buy and hold strategy: the R-CVaR delivers better returns than a Markowitz portfolio, even when those performances are measured with a mean–variance metric.

## Introduction

A key investment decision for institutional investors is the selection of an optimal asset mix with regard to their pre-defined objectives (Brown et al. 2010; CGFS 2007). The asset mix needs to reflect the desired trade-off between risk and reward (Markowitz 1952) over a certain time horizon and to achieve a satisfactory level of portfolio diversification (Black 1976).

Many authors (Jondeau and Rockinger 2019; Pedraza 2015) investigate institutional investors making long-term portfolio allocation. This paper focuses on Italian Foundations of Banking Origin (FBOs), which are a specific category of institutional investors acting on very long time horizons (i.e., they “invest in perpetuity”). As with endowments, the question for FBOs is how to build and manage a financial portfolio that not only meets short-term needs but also features an investment strategy which complies with legal constraints and, consistently with their mission, meets the need to preserve capital (Pattarin 2018). FBOs are in fact private, independent, non-profit entities, supervised by the Italian Ministry of Economy and Finance (MEF), supporting the social, cultural, political, and economic development of a country (Rangone 2012).^1^ FBOs were set up in the early 1990s by the “Amato Law”,^2^ which privatized Savings Banks and converted them to Foundations. The “Amato Law” required FBOs to maintain the majority ownership of the joint stock Savings Banks, and separated banking functions from philanthropic activities. FBOs have retained part ownership of the most important Italian banks, and although their shareholdings have fallen significantly over the years they remain major shareholders in large Italian financial institutions. In terms of mandate, the 2015 “Protocollo ACRI-MEF”, a Memorandum of Understanding (MoU) between the Associazione Casse di Risparmio Italiane (ACRI) and MEF, laid down that FBOs should not utilize more than a third of total assets, directly or indirectly, on a single-subject investment, using “fair value” methodologies to evaluate the exposure and composition of these assets.

FBOs are the main philanthropic organizations in Italy, and are bound by law to work and expand the charity sector (Minguzzi et al. 2019). FBOs sponsor infrastructure and healthcare projects, social benefit programs, volunteering activities, local community projects, education, culture, art and scientific research. In recent years, they have made total grants of an average of about 1 billion euro per year.^3^ Each FBO operates in its own area, or province or region in which it is based, and also takes part in numerous national and international initiatives coordinated by ACRI. FBOs were particularly important during the Covid-19 pandemic when strong prevention measures were enforced by the government. FBOs mobilized to respond to the needs of their local areas and support economic recovery and scientific research.

Currently, there are 86 Italian Banking Foundations, which vary in size, origin and local activity. At the end of 2020, FBOs’ total endowment funds amounted to 39.7 billion euro, equal to 86% of their liabilities.^4^ Their assets amounted to about 46 billion euros. In terms of composition, FBO assets are 95.1% financial assets and 4.7% movable and immovable assets.

FBOs award philanthropic grants using financial resources deriving from income generated by capital investments. Some income is used for capital management, and as noted above, some investments are in banking activities while others are medium-long term investments, often made in the sectors which are already receiving public subsidies. Such funds are offered, in synergy with Italian government institutions, to social housing projects, small and medium-sized enterprises (SMEs), technological research and other types of infrastructure. Further, some funds finance companies operating in strategic areas such as motorways, airports and also state controlled CDP (Cassa Depositi e Prestiti)^5^ for strategic development projects. Thanks to their sizeable assets,^6^ FBOs can also provide grants for social innovation projects (Maiolini 2017).

FBOs disbursed 949.9 million euro in 2020, and the 2020 figure corresponds to a 2.4% disbursement rate on the average patrimony of all FBOs. The number of initiatives funded was 19,528 in 2020.^7^ It is clear that FBOs play a pivotal role in supporting social impact investments as well as economic and financial growth. Their challenge is to fulfill their higher mission as well as realize higher risk-adjusted returns while striking a balance between risk and potential returns. Decisions on asset allocation and investment returns are fundamental to FBO institutional objectives.

Although the role of FBOs is growing internationally (Lecy and Van Slyke 2013), to our knowledge few studies have to date investigated their portfolio construction strategies. In the light of the importance of social value creation, and stakeholder and community management of FBOs, we use Markowitz’s model (Markowitz 1959) and a robust portfolio optimization procedure minimizing the Conditional VaR (CVaR) proposed by Grossi and Laurini (2019) to examine a possible asset management solution which takes the need to preserve capital into account. The classical Markowitz model and the Robust CVaR (R-CVaR) are compared under the constraints imposed by the “Protocollo ACRI-MEF” and the particular asset management characteristics of FBOs. We first identify an optimal asset allocation. We then use a monthly Buy and Hold (B&H) strategy (i.e., a long-term passive strategy in which investors keep a relatively stable portfolio over time, regardless of short-term fluctuations) to analyse potential differences in returns and risk exposure between the Markowitz model and the R-CVaR approach. The well-known Markowitz portfolio allocation model, based on expected returns and their covariance, has been criticized for many reasons (e.g. Michaud 1989). Financial returns have heavier than Gaussian tails so the covariance of returns could provide a loose quantification of the effective risk. It is also sensitive to small changes in either the expected returns or their correlation, often leading to irrelevant portfolio allocations.

This paper contributes to the existing literature as follows. As discussed in Sect. 3, previous research usually shows results for a fixed level of risk aversion, while here we consider several different risk profiles, described by different parameters of risk aversion following an innovative approach to Expected Shortfall (R-CVaR). Our results range, in fact, from a conservative “expected return at minimum risk” to the much riskier “expected return at maximum risk”, considering a robust tail risk measure as the R-CVaR.

The paper is organized as follows. Section 2 briefly describes the role and process of reform of FBOs. Section 3 presents a literature review on FBOs and the importance of their asset allocation strategy, which motivates the application of a robust portfolio optimization approach. Sections 4, 5 and 6 describe the dataset, the model we use for portfolio optimization with CVaR, and show the empirical findings. Sections 7 and 8 present discussion and conclusions.

## The role and process of reform of FBOs

This section briefly describes the reform of FBOs’ role and functions. FBOs in Italy today are the outcome of a long process of reform of the banking system. In the early nineteenth century, Savings Banks were founded and operated in two areas: (1) credit and (2) charity in their local communities. After the 1st and 2nd Directives (EEC Directive 77/780 and EEC Directive 89/646) on credit, concerning freedom of establishment and banking de-specialisation and the adoption of the "Amato Law” and its implementation decrees, Savings Banks transferred their banking activities to ad-hoc joint-stock banking companies, the new Savings Banks. The original Savings Banks were converted into foundations which took over all the social and charitable functions laid down in Savings Bank statutes.

Originally, the foundations were controlled by the few regulations outlined in Legislative Decree No. 356/90, which implemented the principles laid down in Legislative Decree No. 218/90. Until 1994, the foundations were required to maintain majority ownership of the joint-stock Savings Banks. But Law No. 474 of July 30, 1994, and the Ministry of the Treasury Directive of November 18, 1994, repealed this requirement and tax incentives were introduced to encourage the foundations to relinquish their shareholdings.

Under Law No. 461 of December 26, 1998, the “Ciampi Law”, and the subsequent Implementation Decree, No. 153/99, the Italian parliament created the conditions for the completion of the restructuring process of the banking sector and revised the civil and tax laws relating to FBOs, recognising them as private entities under legal jurisdiction. With the approval of specific legislation by the former Ministry of the Treasury, now MEF, FBO became private, non-profit and autonomous entities. The “Ciampi Law” also required that FBO relinquish control of the Savings Banks, and to encourage this, included a provision for the temporary suspension of Capital Gains Tax on the sale of shares. This tax measure was initially in place for four years but was extended until 31 December 2005 by the Implementation Decree of 15 June 2003. The MoU between ACRI and MEF was signed on 22 April 2015 and aimed to optimise the combination of foundations' profitability objectives and their possible risks. Until 2015, FBOs consistently supported Italian banks, showing a concentration of their resources in bank equity capital (Ayadi et al. 2009; Moscariello 2012). However, the financial crisis of 2008–2009 had a huge negative impact on the FBOs’ investment returns and, consequently, on the grants awarded in areas in which foundations operate (Filtri and Guglielmi 2012).^8^ The MoU therefore agreed that FBOs should not utilise more than a third of their total assets, directly or indirectly, on a single-subject investment, using “fair value” to evaluate the exposure and composition of their assets. FBOs were allowed three years from the date of the MoU to reduce the amount of excess risk exposure where trading of financial instruments on financial markets was involved, and five years where financial markets were not involved. With regard to their current role in banks, the FBOs have the same financial, economic and administrative rights as other shareholders. They are not required to make agreements with trade unions. No representative of a FBO can be nominated for a position on any of the controlling bodies of the related savings bank or any other savings bank. Current legislation in fact lays down complete incompatibility between the administration of FBOs and that of savings banks. Rather, FBOs promote social utility and economic development and they pursue their aims by using the profits deriving from financial investments in order to contribute to socio-economic development through the financing of social projects. It follows that the impact of FBO’s activities is measured in both financial and social terms.

## FBOs: a literature review

Foundations of Banking Origin administer, preserve and increase their assets in order to support the social, cultural, political, and economic development of the area of traditional operations (Barbetta 1999, 2013; Leardini et al. 2014; Ricciuti and Turrini 2018). They operate mainly by awarding contributions to projects and initiatives and, in order be effective, need to contribute to meeting the needs of the area according to a defined plan. So FBOs identify sectors of interest following mainly multi-year planning criteria, through planning, implementing and financing intervention programs and projects. Italian FBOs are however currently changing the ways of intervening in local and national communities. Awarding grants is only one of the many available mechanisms, and new ways, such as investing in social impact bonds, are currently undergoing experimentation by some FBOs (Salamon 2014).

FBOs have many resources available, and in the light of their objectives, these need to be deployed in prudent and profitable investments. One important investment decision is thus the selection of an optimal portfolio asset mix. FBOs use income from their investments to pursue their institutional mission, which usually consists of providing support to different collective-interest sectors (e.g., art and culture, education, research, social assistance, charity, sport, public health) through projects implemented directly or exclusively by private or public non-profit entities. FBOs are able to provide support thanks to the reserve funds accrued over the previous years.

Asset allocation policy is closely linked to specific activities and roles, for institutional investors in general and for FBOs in particular. It is therefore important to describe FBO activity, their role and governance structure, as well as legal constraints in place. The existing literature on FBOs mainly analyses governance aspects. Boesso et al. (2015) investigate the relationship between non-profit foundations’ choice of philanthropic strategy and sound governance. The specific role of the board in strategic planning and in determining effectiveness of activities has also been analysed (Green and Griesinger 1996). Several studies, including Brown (2007), examine how non-profit boards’ performance can affect organizational performance. Fredette and Bradshaw (2012) investigate how the institutional and social context affects boards. Gabrielsson and Huse (2004) examine changes in board composition and action. Many researchers (Brown and Iverson 2004; De Andrés-Alonso et al. 2010; Porter and Kramer 1999) study the relationship between foundation governance and formulation of strategy. Zimmermann and Stevens (2008) identify several best practices for non-profit boards, including the separation of board and staff duties. Siciliano (2008) finds that strong leaders in non-profit organizations enhance the directors’ active role in strategy and leadership stability.

Despite the importance of the topic, to our knowledge, there is little literature analysing the asset allocation strategy of FBOs. However, asset allocation is a very important issue because it accounts for a large part of the variability in the return on an investor's portfolio (Sharpe 1992). In general, investors allocate their wealth following strategies aimed at obtaining high returns, compatibly with their own risk tolerance, and objectives that they either wish or are obliged to achieve (Ibbotson and Kaplan 2000; Lerner et al. 2007). Some studies (Hensel et al. 1991; Xiong et al. 2010) focus on the importance of asset allocation policy in determining performance. In particular, Ibbotson (2010) finds that variation in the returns comes from: (1) financial market movements, (2) the incremental return from the asset allocation policy of the specific portfolio and (3) the active return (the alpha) from timing, selection, and fees. Other studies (e.g., Bessler et al. 2021) analyze different asset allocation models and compare their portfolio performance. Other researchers (Leibowitz and Kogelman 1991; Jacquier and Marcus, 2001) focus on the relationship between the market and portfolio volatility and the asset allocation models. Portfolio theory is crucial to risk management and is based on efficient diversification (Jacquier and Marcus 2001). Efficient diversification, as widely recognised, is linked to asset correlation and covariance structure, which varies over time (Chow et al. 1999; Longin and Solnik 1995; Solnik et al. 1996). Markowitz (1959) developed the pioneer portfolio allocation approach, based on expected returns and their covariance. However, many studies, including Michaud (1989), criticize the use of variance as a risk measure. Variance is estimated using all data, but some of the data do not give relevant information (Grossi and Laurini 2011). Moreover, variance is a proper risk measure for Gaussian distributions (or “normal” distribution), but it is widely recognised that financial returns have tails which are often skewed (Casarin and Billio 2007) and are heavier (De Donno et al. 2019; Nolan 2014; Stoyanov et al. 2006) than Gaussian tails. Heavy losses have the nefarious consequence of permanently reducing capital and its growth trajectory over time, which is the ultimate goal of investors. In other words, non-Gaussian distributions with heavy tails highlight what is termed the “ergodicity problem” in economics (Peters 2019). Alexander and Baptista (2002) also criticize the Markowitz model because within the set of optimal portfolios it yields certain combinations of assets which would be irrelevant for a financial investor. For these reasons and since measures of tail risk have an important role in optimization under uncertainty, Value at Risk (VaR) has been widely used in literature and in practice. However, VaR also suffers from being unstable and difficult to work with numerically when losses are not “normally” distributed (Artzner et al. 1999). An alternative measure able to quantify the losses that might be encountered in the tail, Conditional VaR (CVaR),^9^ is thus more usually adopted (Bogentoft et al. 2001; Ferstl and Weissensteiner 2011; Gulpinar and Pachamanova 2013; Mulvey and Erkan 2006). CVaR is consistent with VaR in yielding the same results for normal distributions (Rockafellar and Uryasev 2002). It is also the case that for portfolios with normal distributions, working with CVaR, VaR, or minimum variance (Markowitz 1952) does not yield very different results.

The CVaR minimization formula was first developed by Rockafellar and Uryasev (2000). However, the optimization procedure used to minimize CVaR requires a numerical routine which is not robust to the presence of extreme returns (Huang et al. 2008). Moreover, since the optimization is based on few observations, it is possible that sub-optimal portfolios could be selected (Gotoh et al. 2013). Chen et al. (2015) propose different measures of risk, but these rely on diversification via the Herfindahl index which is sensitive to outliers. Trzpiot and Majewska (2008) analyse the effect of the application of different robust estimators of risk to efficient frontiers. Hellström (2000) and Maillet and Merlin (2009) develop methods able to identify and correct outliers and robust estimators of portfolio weights. Welsch and Zhou (2007) introduce a robust version of the covariance matrix replacing the classical covariance matrix in the optimization procedure. Ledoit and Wolf (2004) develop an estimator of covariance matrix of portfolio assets based on shrinkage. Some researchers (DeMiguel and Nogales 2009; Lauprete et al. 2002) propose a single step optimization approach based on robust estimators of the risk measure. Benati (2015) suggests the use of median instead of the mean as the estimator of the return in portfolio optimization. Scutellà and Recchia (2013) examine the properties of robust methods, with application to the estimation of risk measures, but some of their results are inconclusive when compared with other methods. Kremer et al. (2020) present a portfolio optimization framework to solve the mean–variance portfolio problem with a sorted ℓ_1_ regularization, named “SLOPE”, and linear constraints on the asset weights, which makes it possible to group constituents with similar correlation properties and the same risk factor exposures. Grossi and Laurini (2011) propose a robust estimator which weights data using a forward search approach (see also Grossi and Laurini 2009). In this paper we use an optimal portfolio allocation method, developed by Grossi and Laurini (2019), which is based on the robust estimation of the input parameters through a forward search with the allocation conducted minimizing the CVaR and which shows many advantages and outperforms both robust and non-robust alternatives. We compare this approach with the Markowitz model, using a B&H strategy.

## Data

FBOs’ portfolio performance can to a large extent be explained by their strategic asset allocation decisions. In recent years, FBOs have gradually diversified their investments and selected asset classes characterized by different levels of risk and return. In order to achieve high returns relative to traditional stock and bond investments, they have, like other institutional investors, including endowments (Lerner et al. 2008; Merton 1971), increased investments in illiquid alternative assets, such as venture capital, real estate, and art. Many authors (Ang et al. 2014; Dimmock et al. 2019) show, in fact, that sophisticated investors, like FBOs, can diversify wealth by investing in multiple alternative asset funds with automatic liquidity events staggered over time. An empirical examination of liquidity diversification is carried out by Robinson and Sensoy (2016). However, FBO asset allocation remains, on the whole, prudent, so that capital is safeguarded over time and there is a stream of income to provide for grants to be awarded.

In order to analyse FBO asset management choices, we examine their investments across the following asset classes: equity, fixed income, real estate, venture capital and art. We initially consider 100 assets. The daily returns are obtained from Thomson Reuters Datastream, covering the period from January 2009 to December 2018. After cleaning to obtain a sufficiently long time series, our final sample includes 73 assets, of which 60 are equities, 5 are fixed incomes, 3 are real estates, 2 are alternative assets (i.e., venture capital) and 3 assets belong to the art sector. We compute the simple monthly returns for the available data, which yields a clearer mathematical formulation of portfolio performances, as simple returns aggregate across assets. Table 1 shows the descriptive statistics of the dataset. It is worth noting that almost all asset classes have negative skewness (implying, on average, large impact of extreme losses) and all asset classes have fat tails, as they all present kurtosis bigger than 3. It is difficult to see attractive asset classes looking at the aggregate values in Table 1. From our data, Real Estate assets appear to offer a good outlet using “standard” descriptive measures, but as described below, their overall weight in the portfolio depends on the risk profile.

**Table 1:** Descriptive statistics of the dataset

Dataset	N	µ	σ	Med	Min	Max	Skew	Excess-kurt
Fixed income	5	0.004	0.018	0.005	− 0.089	0.081	− 0.215	2.530
Real Estate	3	0.008	0.052	0.005	− 0.178	0.181	0.143	1.115
Alternatives	2	0.014	0.062	0.019	− 0.184	0.253	− 0.106	1.103
Art	3	0.011	0.157	− 0.004	− 0.394	1.204	3.057	19.845
Equity, of which:	60	0.006	0.082	0.009	− 0.693	0.567	− 0.495	5.019
Banking equity	9	− 0.001	0.116	0.005	− 0.431	0.504	− 0.215	1.101
Other equity	51	0.007	0.074	0.009	− 0.693	0.567	− 0.560	6.632

This table reports descriptive summary statistics for the 73 assets considered in the analysis: 60 are equity assets (9 banking equities and 51 other equity); 5 are fixed incomes; 3 are real estate assets; 2 are alternative assets; and 3 are art assets. Reported are for the daily data: the number of constituents (*N*), the mean (*µ*), the standard deviation (*σ*), the median (*med*), the minimum (*min*), the maximum (*max*), the skewness (*skew*), and the excess of kurtosis (*excess-kurt*), i.e., the difference between the sample kurtosis and 3

The asset class “Equity” includes single stock, belonging to different economic sectors (i.e., Banks; Travel & Leisure; Automobiles & Parts; Telecommunications; Construction & Materials; Insurance; Media; Basic Resources; Food & Beverage; Real Estate; Chemicals; Health Care; Industrial Goods & Services; Utilities; Financial Services; Retail, Personal & Household Services; Technology; Oil & Gas) and main European equity indexes. Since FBOs are mainly active in Italy, the Italian major stocks (top 40 by market capitalization) are included in our analysis. The main European bond indexes and World Real Estate, Venture capital and Art Indexes are selected.^10^

In order to define the portfolio asset mix, we impose a set of constraints consistent with the average portfolio composition of the main FBOs. In other words, the constraints are determined considering the specific activity of FBOs and their regulation, with particular reference to the 2015 MoU specification that foundations should not utilize more than a third of their total assets, directly or indirectly, for a single-subject investment, using “fair value” to evaluate the exposure and composition of the said assets. To represent the above constraint, as well as sensible targets for FBOs management, we use the following ranges of weights (W) for which an asset class can be selected:

*Equities*: min 15%—max 70% (cannot include more than 33% of banks);

*Banks*: min 15%—max 33%;

*Bonds*: min 10%—max 50%;

*Real estates*: min 2%—max 5%;

*Arts*: min 2%—max 10%;

*Alternatives*: min 2%—max 50%.

For an interpretation of these ranges, take *Alternatives* as an example. The weight of assets in the Alternatives class needs to be such that at least 2% of the portfolio has assets from this asset class, with a maximum of 50%. Note two additional constraints. First, non-short selling is considered, as it is often compulsory in regulated markets. Second, only a part of total cash is usually used to build the portfolio, often about 60–70% in Italian FBOs. Since we also use the budget allocation constraint (i.e., sum of the weights equal to one), all allocations should be “rescaled” suitably. Since we aim to suggest an innovative asset allocation strategy for risk averse investors who operate on very long-time horizons, such as Italian FBOs, we first identify an optimal portfolio asset allocation by using Markowitz’s model and the R-CVaR approach developed by Grossi and Laurini (2019). Indeed, the well-known Markowitz portfolio allocation model, based on expected returns and their covariance, has been criticized for many reasons (e.g. Michaud 1989). Financial returns have heavier than Gaussian tails so the covariance of returns could provide a loose quantification of the effective risk. Moreover, the Markowitz approach is sensitive to small changes in either the expected returns or their correlation, and can often yield irrelevant portfolio allocations.

We thus compare the two methods, identifying potential differences in returns and risk exposure, on a B&H strategy, a long-term, passive strategy where returns are maximised by minimizing buying and selling and where investors keep a relatively stable portfolio over time, regardless of short-term fluctuations.

Unlike many studies cited in Sect. 3, our results are presented for several risk profiles, described by different parameters of risk aversion. Our study in fact ranges from a conservative “expected return at minimum risk” to the much riskier “expected return at maximum risk”. We describe the R-CVaR approach in the following Section.

## The model for portfolio optimization with CVaR

Our analysis aims to compare the Markowitz model (Markowitz 1959) and a R-CVaR approach (Grossi and Laurini 2019) in order to identify an efficient asset allocation strategy for Italian FBOs. The optimal portfolio allocation method (R-CVaR) we adopt is based on the robust estimation of the input parameters through a forward search with the allocation conducted minimizing the CVaR. It uses, in fact, weighted estimators of the input parameters (i.e., portfolio returns and their riskiness), and thus balances the influence of extreme returns on the allocation weights, and obtains more reliable portfolios. This is the main contribution of this approach.

### Review of the mean–variance Markowitz approach

In order to present mathematical details for the portfolio optimization with CVaR, we suppose that there are *N* risky assets, whose prices for *T* periods are *p*_*it*_, *t* = 1,…, *T*, *i* = 1,…, *N* and *x* = (*x*_1_,…, *x*_*N*_)′ is the vector of portfolio weights (the allocation weights). We start by introducing the classical mean–variance approach of Markowitz following indications given by Broadie (1993).

The asset returns are given by a matrix Y = (*y*_1_*,*…, *y*_*N*_), where *y*_*i*_ = (*y*_*i1*_*,*…*, y*_*it*_*,*…*, y*_*iT*_)′ and *y*_*it*_ = ln(*p*_*it*_*/p*_*it−1*_) ≈ (*p*_*it*_*/p*_*it−1*_) − 1 with expected returns given by a N × 1 vector μ and N × N expected covariance matrix ∑. The expected return and variance of the portfolio can be expressed as follows: *μ*_*p*_ = *x′μ* and σ^2^_p_ = *x′ ∑x*, respectively. Our exposition does not include any risk-free asset.

The formula for the classical mean–variance optimization problem is the following:

1 $$ \mathop {\max }\limits_{x} (x^{\prime } \mu - \lambda x^{\prime } \sum x) $$

for a given level of risk aversion λ (i.e., λ is an arbitrary parameter selected by the user). It is subject to the constraints of *x* ≥ 0 (i.e., all the weights are strictly non-negative) and *x*′ι_*N*_ = 1, where ι_*N*_ is a N × 1 vector of ones. We impose the constraint of no short selling (*x* ≥ 0) given that many funds and institutional investors, including FBOs, are not allowed to sell stocks short. Notice that the restriction *x* ≥ 0 implies that the solution to Problem (1) is non analytical and numerical routines are necessary.

Decreasing λ from a large number to zero, for each specified value of λ the optimization problem is solved, and the efficient frontier is built. Take as an example the vector of expected returns and covariance matrix of Broadie (1993) given, respectively, as

$$\mu =\left(0.006, 0.01, 0.014, 0.018, 0.022\right)$$

$$\Sigma =\left[\begin{array}{c}\begin{array}{ccccc}0.0072250& 0.00204& 0.0024225& 0.002295& 0.00255\\ 0.0020400& 0.00640& 0.0022800& 0.002160& 0.00240\\ 0.0024225& 0.00228& 0.0090250& 0.002565& 0.00285\\ 0.0022950& 0.00216& 0.0025650& 0.008100& 0.00270\\ 0.0025500& 0.00240& 0.0028500& 0.002700& 0.01000\end{array}\end{array}\right]$$

**Fig. 1:**
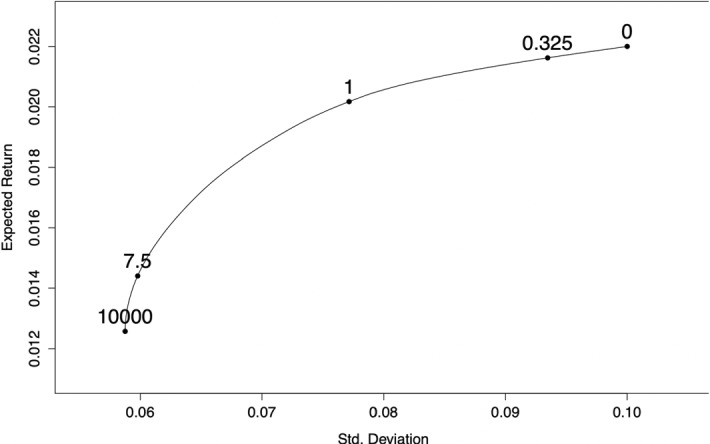
Efficient frontier using $\mu$ and σ of Broadie (1993). Some values of λ are indicated along the frontier. Large λ = less risky asset allocation; Small λ = riskier asset allocations

For this choice of $\mu\,and\,\Sigma$, the efficient frontier is found by choosing a set of weights *x* which satisfy Eq. (1) for a range of values of $\lambda \in$[0, $\infty$). A sketch of the frontier with the choice of input parameters given above is shown in Fig. 1. In our setting, the minimum risk portfolio implies a high value of λ. In practice, any choice of $\lambda$ big enough (Broadie 1993, takes $\lambda$ = 10,000) provides a combination risk/reward which yields the minimum risk portfolio on the efficient frontier.

### Asset allocation with minimum CVaR

Following Grossi and Laurini (2019), this paper makes some substitutions in mean–variance optimization: we use CVaR as a measure of risk instead of variance and we express the optimization problem using the risk aversion formulation, in order to explore the results for a variety of values of λ. This is an important innovation compared to other studies; the very recent work of Kremer et al. (2020), for example, uses minimum variance portfolios, which makes any comparison of little practical relevance.

In the following exposition we present all changes from the Markowitz approach. From a conceptual viewpoint there is a minor adjustment: the measure of risk is the CVaR instead of the variance (or standard deviation). For a variable *Y* generating the multivariate set of returns, we select each component with $Z= {\sum }_{i=1}^{N}{x}_{i}{Y}_{i}$, so that –Z represents the distribution of the losses. The CVaR of order α ∈ (0, 1) of the random variable Z is

$${\rm CVaR}_{\alpha }\left(Z\right)= \frac{1}{\alpha } \int\limits_{0}^{\alpha}{VaR}_{u}\left(Z\right)du$$

where ${VaR}_{\alpha } \left(Z\right)=$–inf{*z*: Pr(Z ≤ *z*) ≥ α} is the VaR of Z of order α.

Rockafellar and Uryasev (2000) prove that a convenient representation for computing jointly VaR and CVaR is to use the function

2 $${G}_{\alpha }\left(s, Z\right)=-s-\frac{1}{\alpha }E\left[{\left(s+Z\right)}^{+}\right]$$

which is jointly convex in (*s*, Z) for any α (with s ∈ ℝ and Z in the space of the random variable induced by the linear combination of Y). We use the notation (*b*)^+^  = max(0, *b*). Therefore, CVaR is given by

$$ {\text{CVaR}}_{\alpha } \left( Z \right) = \mathop {\max }\limits_{{s \in {\mathbb{R}}}} G_{\alpha } \left( {s, Z} \right) $$

where under some assumptions, ${\mathrm{VaR}}_{\alpha } \left(Z\right)= {s}^{*}$ is the solution of the maximization problem. Our model takes the maximum of the negative function. This is different, but mathematically equivalent, from the representation of Rockafellar and Uryasev (2000), who consider the minimum of the positive function. Therefore, Problem (1), in the CVaR approach, becomes:

3 $$\left\{\begin{array}{*{20}l}{max}_{x} {x}^{\prime}\mu - {\lambda \mathrm{CVaR}}_{\alpha } \left({x}^{{\prime}}Y\right)= {max}_{x,s} \left\{{x}^{{\prime}}Y- \lambda s-{\lambda \alpha }^{-1} \mathrm{E}\left[{\left(s+ {x}^{{\prime}}Y\right)}^{+}\right]\right\}\\ {\sum {x}_{i}}=1\\ {x}_{i} \ge 0, {\forall }_{i}\end{array}\right.$$

When Problem (3) is solved for a range of values of λ, the efficient frontier is obtained, as proved in Theorem 3 of Krokhmal et al. (2002) and in Fabozzi et al. (2007).

**Fig. 2:**
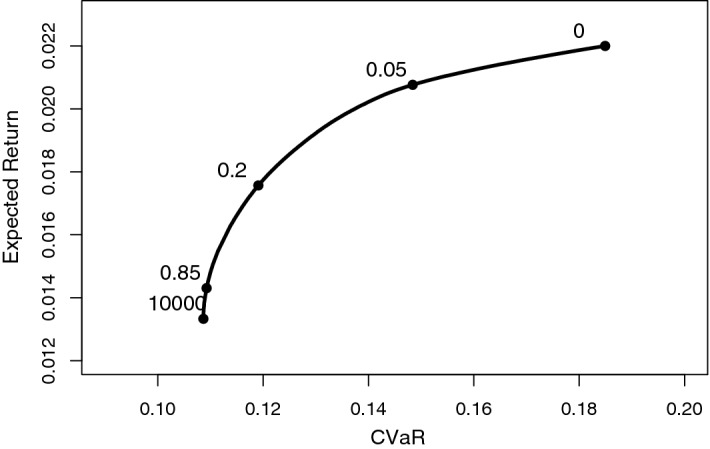
Efficient frontier with the CVaR on the horizontal axis. Some values of λ are indicated along the frontier. Large λ = less risky asset allocation; Small λ = riskier asset allocations

Using the same vector of expected returns, the same covariance matrix and assuming Gaussian returns (for the sake of exposition) and by varying the value of $\lambda \in$[0, $\infty$), we build the efficient frontier (See Fig. 2). Comparing this frontier with the Markowitz model, the abscissa shifts because the unit of measurement of risk is different. For this reason, the values of $\lambda$ in the two approaches are not directly comparable.

Many existing papers (see Sect. 3) use only the minimum risk portfolio, and a single large value of the risk aversion coefficient is used. Here, on the other hand, the algorithm could be evaluated for any values of risk aversion coefficients. Our results focus on the two most extremes values of λ, and highlight differences for low risk compared to risky allocations.

## Results

Consistently with studies finding that initial strategic asset allocation choices are the main determinants of portfolio performance (e.g., Brinson et al. 1986, 1991)^11^ and in the light of FBO characteristics, including that they have an unlimited investment horizon, we first identify an optimal asset allocation by using the Markowitz model and the Robust CVaR approach, and compare the two approaches applied to a monthly B&H strategy.

Tables 2, 3, 4 and 5 report the portfolio performance obtained using the Markowitz model (Tables 2 and 3) and the Robust CVaR approach (Tables 4 and 5). Two risk profiles are considered in the performance: “maximum reward” (Tables 2 and 4) and “minimum risk” (Tables 3 and 5). These two risk profiles set the risk aversion parameters at the extreme points of the efficient frontier.

**Table 2:** Markowitz model—Portfolio performance and descriptive statistics of portfolio assets (maximum reward profile)

Annual compound return	2016	2017	2018					
	− 0.018	0.079	− 0.016					
Sortino ratio	0.168							
Dataset	W	µ	σ	Med	Min	Max	Skew	Excess-kurt
MSCI World Real estate	0.02	0.008	0.045	0.007	− 0.126	0.181	0.290	1.909
Post Venture Capital Index	0.02	0.009	0.060	0.013	− 0.184	0.253	0.137	2.383
Madrid Graphic Arts	0.02	0.012	0.061	0.018	− 0.140	0.125	− 0.318	− 0.435
DAX Index	0.076	0.010	0.038	0.012	− 0.119	0.121	− 0.117	0.797
European Monetary Union (EMU) Benchmark 10 years DS Govt. Index	0.5	0.004	0.0167	0.005	− 0.032	0.052	0.184	− 0.227
BPER Bank	0.029	− 0.007	0.124	− 0.002	− 0.378	0.289	− 0.177	0.468
SNAM	0.080	0.003	0.047	0.009	− 0.180	0.101	− 0.784	1.403
Davide Campari Milano	0.042	0.016	0.056	0.014	− 0.106	0.192	0.196	0.320
FTSE Europe Banks	0.119	0.005	0.067	0.007	− 0.187	0.311	0.411	3.317
FTSE Europe Health Care	0.094	0.011	0.035	0.010	− 0.079	0.093	− 0.104	− 0.096

This table shows annual compound returns, Sortino ratio, and descriptive summary statistics for the portfolio selected using the Markowitz model and adopting a maximum reward profile. The table reports: weight of the asset in the portfolio (*W*) and for the monthly average of simple returns: the mean (*µ*), the standard deviation (*σ*), the median (*med*), the minimum (*min*), the maximum (*max*), the skewness (*skew*), and the excess of kurtosis (*excess-kurt*)

**Table 3:** Markowitz model—Portfolio performance and descriptive statistics of portfolio assets (minimum risk)

Annual compound return	2016	2017	2018					
	− 0.238	0.215	− 0.082					
Sortino ratio	− 0.160							
Dataset	W	µ	Σ	Med	Min	Max	Skew	Excess− kurt
MSCI Europe Real Estate	0.02	0.011	0.049	0.004	− 0.105	0.173	0.475	0.651
TR Venture Capital Index	0.16	0.019	0.063	0.023	− 0.161	0.164	− 0.342	0.145
Artprice Index	0.02	0.011	0.188	− 0.012	− 0.394	1.203	2.712	13.904
Italy Benchmark 10 years DS Govt. Index	0.1	0.004	0.026	0.008	− 0.089	0.081	− 0.475	2.007
Banca Generali	0.333	0.018	0.108	0.027	− 0.272	0.504	0.530	3.785
Azimut Holding	0.367	0.010	0.098	0.016	− 0.303	0.285	− 0.263	0.167

This table shows annual compound returns, Sortino ratio, and descriptive summary statistics for the portfolio selected using the Markowitz model and adopting a minimum risk. The table reports: weight of the asset in the portfolio (*W*) and for the monthly average of simple returns: the mean (*µ*), the standard deviation (*σ*), the median (*med*), the minimum (*min*), the maximum (*max*), the skewness (*skew*), and the excess of kurtosis (*excess-kurt*)

**Table 4:** R-CVaR model—Portfolio performance and descriptive statistics of portfolio assets (maximum reward profile)

Annual compound return	2016	2017	2018					
	− 0.016	0.119	− 0.029					
Sortino ratio	0.183							
Dataset	W	µ	Σ	Med	Min	Max	Skew	Excess− kurt
MSCI world real estate	0.02	0.008	0.045	0.007	− 0.126	0.181	0.290	1.909
Post venture capital index	0.02	0.009	0.060	0.0132	− 0.184	0.253	0.137	2.383
Artprice Index	0.028	0.010	0.188	− 0.012	− 0.394	1.203	2.712	13.904
EMU benchmark 10 Years DS Govt. Index	0.505	0.004	0.017	0.005	− 0.032	0.052	0.184	− 0.227
Davide Campari Milano	0.181	0.016	0.056	0.0138	− 0.106	0.192	0.196	0.320
Diasorin	0.065	0.014	0.066	0.010	− 0.165	0.177	0.002	0.081
Banca Generali	0.145	0.018	0.108	0.027	− 0.272	0.504	0.530	3.785
FTSE Europe Media	0.036	0.010	0.038	0.010	− 0.085	0.106	0.071	− 0.033

This table shows annual compound returns, Sortino ratio, and descriptive summary statistics for the portfolio selected using the CVaR model and adopting a maximum reward profile. The table reports: weight of the asset in the portfolio (*W*) and for the monthly average of simple returns: the mean (*µ*), the standard deviation (*σ*), the median (*med*), the minimum (*min*), the maximum (*max*), the skewness (*skew*), and the excess of kurtosis (*excess-kurt*)

**Table 5:** R-CVaR model—Portfolio performance and descriptive statistics of portfolio assets (minimum risk)

Annual compound return	2016	2017	2018					
	− 0.097	0.167	− 0.034					
Sortino ratio	0.033							
Dataset	W	µ	Σ	Med	Min	Max	Skew	Excess-kurt
MSCI Europe Real Estate	0.05	0.011	0.049	0.004	− 0.105	0.173	0.475	0.651
TR Venture Capital Index	0.04	0.019	0.063	0.023	− 0.161	0.164	− 0.342	0.145
Artprice Index	0.105	0.010	0.188	− 0.012	− 0.394	1.203	2.712	13.904
EMU Benchmark 10 Years. DS Govt. Index	0.069	0.004	0.017	0.005	− 0.032	0.052	0.184	− 0.227
France Benchmark 10 Years DS Govt. Index	0.041	0.005	0.018	0.006	− 0.039	0.054	− 0.075	0.077
Davide Campari Milano	0.121	0.016	0.056	0.014	− 0.106	0.192	0.196	0.320
A2A	0.032	0.002	0.088	0.009	− 0.243	0.253	− 0.686	0.714
Banca Generali	0.128	0.018	0.110	0.027	− 0.272	0.504	0.530	3.785
FTSE Europe Banks	0.022	0.005	0.067	0.007	− 0.187	0.311	0.411	3.317
FTSE Europe Health Care	0.392	0.011	0.035	0.010	− 0.079	0.093	− 0.104	− 0.096

This table shows annual compound returns, Sortino ratio, and descriptive summary statistics for the portfolio selected using the CVaR model and adopting a minimum risk. The table reports: weight of the asset in the portfolio (*W*) and for the monthly average of simple returns: the mean (*µ*), the standard deviation (*σ*), the median (*med*), the minimum (*min*), the maximum (*max*), the skewness (*skew*), and the excess of kurtosis (*kurt*)

**Fig. 3:**
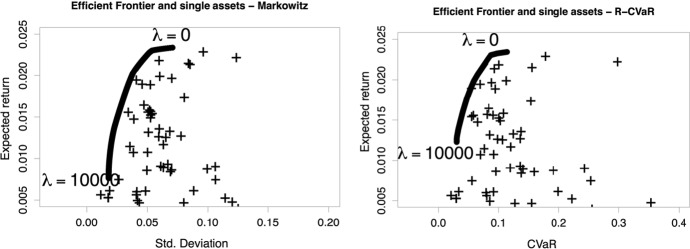
Efficient frontier estimation with Markowitz method (left) and R-CVaR (right). The “ + ” symbols indicate single assets

The efficient frontiers for both Markowitz and R-CVaR approached are sketched with the two boundaries for λ at the extremes of the frontier. In both panels Fig. 3, the single assets are denoted by the “ + ” sign. We recall that small λ denotes more risky asset allocations, and refer to this as the “maximum reward”. Large λ is associated with lower risk allocations, and we refer to this as the “minimum risk”. We also recall that the abscissa in the two panels are not directly comparable as they express risk in different units of measurement.

As specified in Sect. 4, we compute the simple monthly returns for the available data. Portfolio value can be computed with a weighted average of simple returns. With simple returns, the temporal portfolio performance is evaluated with a geometric average.

The Markowitz and the R-CVaR approaches were calibrated on a test sample of monthly returns from January 2009 to December 2015. Performance was then tested on monthly returns using a three-year time horizon from January 2016 to December 2018 on a B&H strategy. The analysis is conducted over a period of three years, because an FBO Strategic Plan, the document that frames overall strategy in support of the local area and defines sectors for intervention, typically refers to this time span. Applied to a B&H strategy, the two approaches, the Markowitz model and R-CVaR, yield different results.

Looking at the portfolio selected by the Markowitz model assuming the maximum risk profile (Table 2), we observe that 50% of the portfolio consists of fixed incomes (i.e., EMU Benchmark 10YR. DS Govt. Index) and about 44% is invested in equity (i.e., more than 21% in European equity indexes, relating to banking and health care sectors, respectively; almost 8% in the German DAX Index; about 15% in Italian stocks). Only 6% of the portfolio is allocated to alternative assets (i.e., real estate,^12^ venture capital and art). Table 3 shows asset allocation selected by the Markowitz model assuming the minimum risk profile. Note that the number of portfolio assets is lower and the geographical area to which they relate is smaller than for maximum risk aversion (e.g., Italy Benchmark 10 years DS Govt. Index instead of EMU Benchmark 10 years DS Govt. Index and MSCI Europe real estate index^13^ instead of MSCI World real estate index versus). Almost 70% of the portfolio consists of equities (i.e., Banca Generali and Azimut Holding), 2% is invested in real estate, 2% in art and 16% consists of venture capital index. Only 1% of the portfolio is allocated to fixed income assets (i.e., Italy benchmark 10 years DS Government Index).

The asset allocation selected by the R-CVaR model assuming the maximum risk profile (Table 4) is characterized by the predominance of fixed incomes (i.e., more than 50% of the portfolio is invested in the EMU Benchmark 10-year DS Govt. Index). Almost 43% is invested in equity, of which more than 39% consists of Italian stocks (including 14.5% of banking stocks) and 3.6% is invested in the FTSE Europe Media Index. Almost 7% of the portfolio is allocated to alternative assets (real estate, venture capital and art).

Looking at the asset mix selected by the R-CVaR approach assuming the minimum risk aversion, we observe greater portfolio diversification than the Markowitz model. Almost 70% of the portfolio is allocated to equity, of which about 41% is European indexes (i.e., FTSE Europe Banks and Health Care) and 28% are Italian stocks (including almost 13% of banking stocks); 11% of the portfolio consists of bond indexes (i.e., EMU Benchmark 10 years Government Index and France Benchmark 10 years Government Index). Finally, almost 20% of the portfolio is allocated to alternative assets, of which 10.5% in the Art sector (i.e., Artprice Index).

**Fig. 4:**
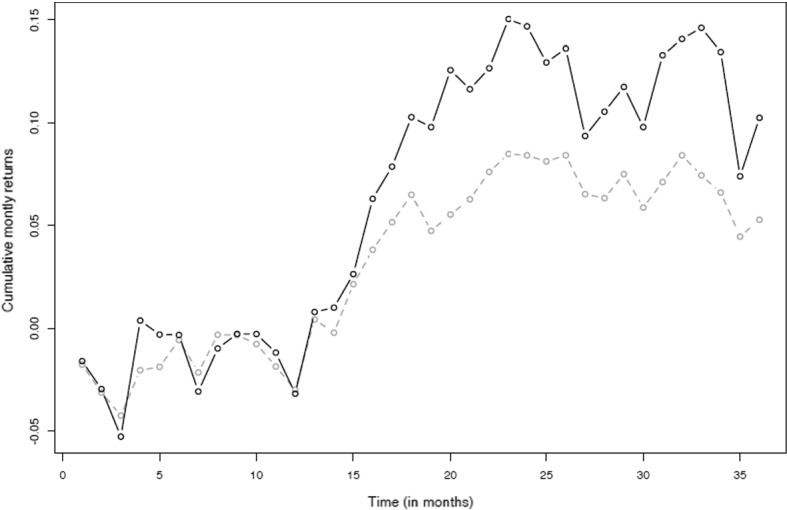
Cumulative monthly returns—“maximum reward profile”. Monthly performances according to Markowitz’s model (grey line) and the R-CVaR optimization model (black line) considering the maximum reward profile

**Fig. 5:**
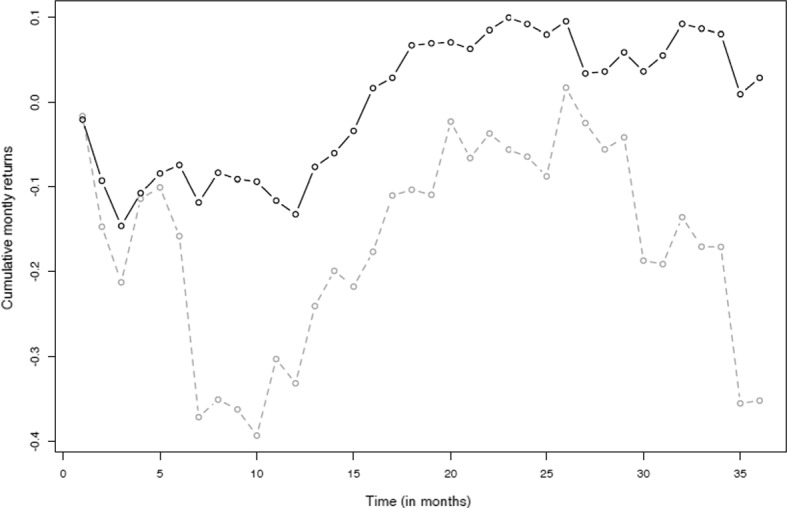
Cumulative monthly returns—“minimum risk”. Monthly performances according to Markowitz’s model (grey line) and the R-CVaR optimization model (black line) considering the minimum risk profile

As described above, the performance of the portfolios identified by the Markowitz and the R-CVaR models is compared in application to a B&H strategy from January 2016 to December 2018. It seems that the R-CVaR model amplifies the positive/negative effect, in terms of cumulative monthly returns, of the Markowitz model. This is shown in Figs. 4 and 5.

Our aim is to compare the two approaches in terms of risk exposure as well as performance. We thus calculate the Sortino ratio, a measure of risk-adjusted performance which uses downside deviation as the measure of risk (i.e., only those returns falling below a user-specified target or required rate of return are considered risky), which is relevant to investors (Harlow and Rao 1989), in this case, FBOs. The target downside deviation is the root-mean-square of the deviations of the realized return’s underperformance from the target return, where all returns above the target return are treated as underperformance of 0. Looking at the Sortino ratio values for the Markowitz model (0.168 and -0.160 assuming maximum and minimum risk aversion, respectively) and the CVaR approach (0.183 and 0.033 assuming the maximum and minimum risk aversion, respectively), the Robust CVaR shows the highest values, indicating that the variability of returns is not concentrated mainly below the minimum considered acceptable by the investor. A low Sortino index on the other hand indicates that the variability is concentrated below the acceptable minimum.

## Discussion

The comparison between the Robust CVaR (Grossi and Laurini 2019) and the Markowitz model shows that the R-CVaR approach allows for more an efficient capital allocation in terms of number of portfolio assets, stock picking (considering the relevant geographical area among other factors) and the weight of assets in the portfolio, consistently with the assumed level of risk aversion. R-CVaR can allow FBOs to plan for more regular and higher cash disbursements to their beneficiaries, compared to allocation based on the classical Markowitz model.

We also find that the R-CVaR delivers better returns that the Markowitz model for a B&H strategy in the medium term, assuming low risk profiles, which is the case for risk averse investors like FOBs. This is a surprising finding. It is however in line with the clear outperformance of B&H during the period 2008–2019 compared to many active asset allocation strategies and Hedge Funds, and with the very strong performance of financial markets during the period of recovery post 2008, partly supported by various Quantitative Easing programs from Central Banks around the globe.

In compliance with Legislative Decree No. 153 of May 17, 1999, FBOs use the income generated by careful management of their investments to provide support to various significant projects of collective interest. It follows that a good robust portfolio optimization model is an important resource for effective FBO asset allocation, consistently with constraints imposed by the 2015 MoU “Protocollo ACRI-MEF”.

## Conclusions

This paper describes an innovative technique of robust portfolio optimization (R-CVaR) which is particularly suitable for investors with a strong aversion to tail losses, such as Italian Foundations of Banking Origin (FBOs). FBOs are important institutions because their mission includes promoting development in their local areas as well as across the entire country. We focus on FBOs, considering specific characteristic that they have in common with other institutional investors, such as endowment funds. To advance their charitable or philanthropic purposes, they invest and use resources drawn from the profits generated by the investments of their considerable assets; they are usually intended to exist in perpetuity and, as such, are regarded as very long-term investors. Since FBOs have an unlimited investment horizon, they are particularly sensitive to capital preservation and avoidance of extreme losses.

FBOs have a detailed regulatory framework with specific investment mandates. In particular, the mandatory constraints introduced by the 2015 ACRI—Ministry of Economy and Finance Memorandum of Understanding are fundamental in determining optimal portfolio decisions for FBOs. In our analysis we include the following asset classes: equity, fixed income, real estate, venture capital, art and cash^14^; and we impose a set of constraints (i.e., weights) for each asset class, in order to reflect legal provisions and requirements. We also apply a non-short selling constraint.

In order to identify an optimal portfolio mix, in the light of the strong aversion to loss of FBOs, we apply the R-CVaR approach developed by Grossi and Laurini (2019) nested within a portfolio diversification framework based on the Markowitz model. We find that R-CVaR allows for more efficient capital allocation. Once the optimal asset mix is identified, we use a B&H strategy and find that R-CVaR again delivers better returns than the Markowitz model. The results of our analysis establish R-CVaR as an optimal asset allocation strategy for particularly risk averse investors, such as endowment funds and FBOs, which at the same time improves their risk-adjusted performance measured by common mean–variance metrics (Sortino Ratio). Overall, we suggest that the use of a new robust portfolio optimization model could make FBO asset allocation more effective. Given the important role played by FBOs in supporting socio-economic growth, our findings should be of interest to decision-making bodies and asset manager of FBOs.

Further research might be needed if a broader set of assets is considered. For example, although it is unlikely, FBOs might consider non-European bond or stocks, for which there is an additional risk related to the exchange rate. The research could also be generalized or adapted to investigate asset classes including security derivatives with non-linear payoff.

## Footnotes

1 As noted by Malesky and Taussig (2009), financial intermediaries and institutional investors are facilitators of economic growth.

2 Italy’s Law No. 218 of July 30, 1990, and the subsequent Legislative Decree No. 356 of November 30, 1990.

3 Source: ACRI, https://www.acri.it.

4 Source: ACRI (2021), Twenty-sixth Annual Report.

5 CDP is the Italian National Promotion Institute (NPI) and Development Finance Institution.

6 Foundations of other types are smaller than FBOs (Hopt et al., 2009).

7 Source: ACRI (2021), Twenty-sixth Annual Report.

8 See Centro Studi e Ricerche di Itinerari Previdenziali, *Investitori istituzionali italiani: iscritti, risorse e gestori dei patrimoni previdenziali per l’anno 2014.*

9 CVaR is also identified as Expected Shortfall (ES).

10 The list of portfolio assets is available on request.

11 Other research (Blake et al., 1999; Ibbotson and Kaplan, 2000) on mutual fund and pension fund investment decisions shows that asset allocation decisions determine variation in returns over time, but it is not relevant in explaining variation returns in the cross-section.

12 The MSCI World Real Estate Index is a free float-adjusted market capitalization index that consists of large and mid-cap equity across 23 Developed Markets countries. All securities in the index are classified in the Real Estate Sector according to the Global Industry Classification Standard (GICS®).

13 The MSCI Europe Real Estate 20/35 Capped Index is designed to measure the performance of the large and mid-cap securities across 15 Developed Markets countries in Europe. All securities in the index are classified in the Real Estate sector as per the Global Industry Classification Standard (GICS®).

14 It is important to note that in our study only a part of the total available assets was used to compose the simulated portfolios.
